# Biological functions of macrophage-derived Wnt5a, and its roles in human diseases

**DOI:** 10.18632/oncotarget.11874

**Published:** 2016-09-06

**Authors:** Yue Shao, Qianqian Zheng, Wei Wang, Na Xin, Xiaowen Song, Chenghai Zhao

**Affiliations:** ^1^ Department of Pathophysiology, College of Basic Medical Science, China Medical University, Shenyang, China

**Keywords:** Wnt5a, β-catenin, macrophage, angiogenesis, lymphangiogenesis

## Abstract

Wnt5a is implicated in development and tissue homeostasis by activating β-catenin-independent pathway. Excessive production of Wnt5a is related to some human diseases. Macrophage recruitment is a character of inflammation and cancer, therefore macrophage-derived Wnt5a is supposed to be a player in these conditions. Actually, macrophage-derived Wnt5a maintains macrophage immune function, stimulates pro-inflammatory cytokine release, and induces angiogenesis and lymphangiogenesis. Furthermore, macrophage-derived Wnt5a is involved in insulin resistance, atherosclerosis and cancer. These findings indicate that macrophage-derived Wnt5a may be a target in the treatment of these diseases. Notably, unlike macrophages, the exact role of macrophage-derived Wnt5a in bacterial infection remains largely unknown.

## INTRODUCTION

Human Wnt family include 19 members of highly conserved secreted proteins, which are critical to embryo development and adult tissue homeostasis [1]. Some Wnt proteins, such as Wnt1 and Wnt3a, activate canonical Wnt pathway. They bind to their membrane receptor Frizzleds (Fzds) and co-receptor lipoprotein receptor-related protein 5/6 (LRP5/6), leading to β-catenin accumulation in the cytoplasm and translocation into the nucleus [2]. However, other proteins, such as Wnt5a and Wnt11, do not trigger Wnt/β-catenin pathway. Generally, pathways stimulated by these proteins are called non-canonical Wnt pathways.

Macrophages are crucial for homeostasis, inflammation and immunity. As professional phagocytic cells, macrophages engulf and degrade cellular debris, dead/damaged cells, foreign substances and microbes. Moreover, macrophages initiate immune response to invading microbes and tissue regeneration upon injury. Macrophages can be found in all organs and take various forms, such as microglia, Kupffer cells, alveolar macrophages, Langerhans cells, histiocytes and others. For a long time, it has been believed that tissue-resident macrophages come from bone marrow derived monocytes (BMDM). However, recently accumulating evidence reveals that most of these tissue-resident macrophages arise from embryonic precursors which reside in these tissues prior to birth [3].

## WNT5A SIGNALING

**Figure 1:**
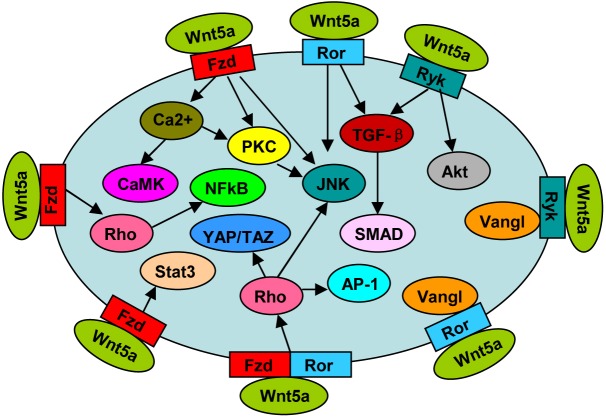
Wnt5a signal pathway

Non-canonical Wnt5a pathways (Figure 1) are usually initiated by the binding of Wnt5a to its receptor Fzds, including Fzd1-5[4–7], and Fzd7 [8, 9]. The intracellular pathways are mediated by some molecules, such as Ca^2+^, CaMKII, PKC and JNK [7, 10]. Ror1/2 are receptor tyrosine kinases, possessing an extracellular cysteine-rich domain (CRD) similar to the Wnt-binding sites of Fzds [11]. Wnt5a-Ror2 activates JNK signaling [12, 13] and TGF-β signaling [14], whereas has no effect on intracellar Ca^2+^ level [6]. Fzds and Ror1/2 can form receptor complexes, which transduce Wnt5a signaling to activate Rho GTPase [8]. Receptor tyrosine kinase Ryk is another Wnt5a receptor [15], involved in the activation of TGF-β-SMAD signaling [16] and AKT signaling [9]. Fzds [5], Ror1/2 [17] and Ryk [18] all mediate Wnt5a-planar cell polarity (PCP) signaling, and both Ror2 [17] and Ryk [18] can form complex with Vangl2, a key component in PCP.

Wnt5a signaling may act on canonical Wnt/β-catenin pathway. To a large extent, the action depends on the receptor type and the cellular context. Wnt5a inhibited Wnt3a-dependent β-catenin accumulation in HelaS3, MKN-1, CHO, and L cells [4]. In these cells, Wnt5a interfered with Wnt3a-induced LRP6 phosphorylation, and competed with Wnt3a for Fzd2 binding [4]. Wnt5a-Fzd2 also suppressed β-catenin expression and activity in hepatocyte [19]. However, in HEK 293 cells, Wnt5a-Ror2 reduced β-catenin-dependent gene expression, but did not affect Wnt3a-induced β-catenin stabilization [6]. Moreover, Wnt5a inhibited β-catenin target gene expression in colon cancer cells via PKC-induced Rorα phosphorylation [20]. Intriguingly, Wnt5a could also activate Wnt/β-catenin pathway, which depended on receptor Fzd4 [6].

## WNT5A EXPRESSION IN MACROPHAGES

Basal Wnt5a expression was observed in peripheral blood mononuclear cell (PBMC) [21], PBMC-derived macrophage [22, 23], bone marrow-derived macrophage (BMDM) [24], alveolar macrophage [22], peritoneal macrophage, microglia [25] and macrophage cell line Raw264.7 [24]. Moreover, Wnt5a receptor Fzd5 expression was also found in macrophages [22–24], suggesting that Wnt5a can affect macrophages in an autocrinal manner. Fzd5 expression in macrophages also indicates that Wnt5a from other cells, such as tumor cells, can exert effects on macrophages in a paracrinal manner. Wnt5a expression in macrophages could be upregulated in response to bacterial stimulation, including LPS [22, 23, 26, 27]. This process depended on Toll-like receptors (TLRs) and NF-κB signaling [22, 26]. IFN-γ synergized with LPS in Wnt5a induction, which could be blocked by anti-inflammatory factor IL-10 [23, 26]. Intriguingly, in murine BMDM and murine microglia there was no upregulation of Wnt5a but Wnt6 [25, 28].

Under the sterile inflammatory conditions, pro-inflammatory cytokines can induce macrophages to secrete Wnt5a. Anti-TNF-α antibody blocked TNF-α-induced Wnt5a expression, whereas had no effect on mycobacteria- or LPS-induced Wnt5a production, suggesting TNF-α-dependent Wnt5a induction is not related to LPS-TLR-NF-κB signaling [22]. IL-1β, IL-6 and CCL2 could also stimulate Wnt5a expression in macrophages [23]. Actually, pro-inflammatory cytokines can induce Wnt5a expression in various cell types. For instance, TNF-α upregulated Wnt5a expression in bone marrow stromal cells (BMSCs) [29], mesenchymal stem cells (MSCs) [30], adipocytes [31], and human dental pulp cells (HDPCs) [32].

Non-inflammatory factors can also stimulate macrophages to overproduce Wnt5a. Oxidized Low density lipoprotein (Ox-LDL), but not native-LDL, induced Wnt5a expression in human macrophages, supporting the notion that macrophage-derived Wnt5a plays a positive role in atherosclerosis [33]. In coculture systems, breast cancer cells and basal cell carcinoma (BCC) cells provoked macrophages to produce Wnt5a [34–36]. In addition, Wnt5a expression was upregulated in hypoxia condition, suggesting that Wnt5a may be involved in the hypoxia-induced macrophage activation [37].

Importantly, macrophage-derived Wnt5a are not only secreted as soluble proteins, it is also delivered to the recipient cells through macrophage-derived exosomes and microvesicles, suggesting that macrophage-derived Wnt5a could have wide range effects [36].

## BIOLOGICAL FUNCTIONS OF MACRO-PHAGE-DERIVED WNT5A

### Maintenance of macrophage immune function

Monocytes can differentiate into macrophages or dendrite cells (DCs) dependent on the stimulator. Under the stimulation of monocyte-colony stimulator factor (GM-CSF), less bone marrow-derived monocytes from Wnt5a monocyte conditional knockout mice differentiated into F4/80^+^ and CD11b^+^ macrophages, compared with those from control mice, demonstrating that Wnt5a is involved in macrophage differentiation[38]. In addition, Wnt5a-negative BMDMs displayed decreased survival, which was related to the reduced expression of anti-apoptotic Bcl2 and Bcl-xl, and the increased expression of pro-apoptotic Bax [24]. Interestingly, β-catenin was also involved in macrophage proliferation and survival [39]. It is unknown whether interactions exist between the two Wnt pathways in macrophages.

Wnt5a inhibition reduced IFN-β and IFN-γ production by macrophages, and the underlying mechanism was related to the decreased IκB kinase β (IKK2) activity, which further resulted in suppressed IκB degradation, and p65 nuclear translocation [24]. As p65 can bind to CD14 enhancer/promoter, Wnt5a pathway blocking also inhibited the expression of CD14 [24], a key component in TLR-dependent immune responses upon microbial infection. Wnt5a suppression also impaired macrophage phagocytosis, accompanied by a decrease in TNF-α and IL-6 production, and an increase in IL-10 secretion [40]. These findings suggest that Wnt5a may be crucial to the maintenance of macrophage immune function.

### Induction of pro-inflammatory mediators

It has been accepted that Wnt5a is a pro-inflammatory factor, based on the finding that Wnt5a induced pro-inflammatory cytokines in a variety of cell types, such as macrophages [23, 37], endothelial cells (ECs) [41], HDPCs [32], BMSCs [29], and synovial fibroblasts [42]. These cytokines include IL-6, IL-1α and IL-1β, which are extensively involved in human inflammatory diseases. Wnt5a also induced chemokines, such as CCL2 and IL-8 [23, 29, 32, 37, 41, 42], indicating that Wnt5a has a potential to recruit macrophages and neutrophils, which amplify inflammatory responses. Several signalings were identified to mediate the induction process, including Ca^2+^/CaMKII [23], Ca^2+^/PKC [41], NFκB [29, 32, 41], and MAPK [29, 32].

Loss and gain of function mice provided evidence that macrophage-derived Wnt5a attributed to adipose tissue inflammation. Expression of TNF-a and IL-6 was remarkably decreased in obese myeloid Wnt5a knockout mice, whereas increased in obese myeloid-restricted Wnt5a overexpression mice [43]. IL-6 treatment restored insulin resistance in obese Wnt5a-deficient mice, suggesting this inflammatory cytokines in adipose tissue promote obesity-induced metabolic dysfunction [43]. Accordingly, *in vitro* studies showed that rWnt5a stimulated macrophages and adipocytes to express IL-6 via intracellular JNK signaling, which could be blocked by Wnt5a antagonist secreted Frizzled-related protein 5 (SFRP5) [44].

Several studies demonstrated that Wnt5a may exert complicated effects on macrophages. Wnt5a and LPS induced pro-inflammatory cyclooxygenase 2 (COX-2) in microglia, respectively, however, Wnt5a inhibited LPS-induced COX-2 upregulation [45]. Moreover, Wnt5a suppressed LPS-induced activation of NF-κB, a key factor in pro-inflammatory cell signaling [46]. These results suggest that Wnt5a may play anti-inflammatory roles in some inflammatory conditions.

### Dual roles in angiogenesis

Early evidence for the involvement of Wnt5a signaling in angiogenesis came from the study on Fzd5 knockout mice, which died in utero due to defective angiogenesis in yolk sac [47]. Wnt5a and its recptor Fzd5 co-expressed in the developing yolk sac, therefore, Fzd5 deficiency-induced angiogenesis defect may be attributed to impaired Wnt5a signaling. Study on endothelial Wntless (Wls) knockout mice further supported the positive role of Wnt5a in angiogenesis. Wls is a transporter for Wnt proteins. These mice exhibited impaired vascularization, accompanied by decreased EC survival and proliferation, and importantly, the angiogenesis defect could be rescued by introduction of Wnt5a [48]. Furthermore, treatment of mice with the non-canonical Wnt inhibitor TNP470 induced similar angiogenesis defect and EC proliferation inhibition to Wls knockout mice [48].

*In vitro* studies directly indicated that Wnt5a overexpression stimulated EC proliferation and survival. Some Wnt5a-target genes were identified, such as matrix metalloproteinase-1 (MMP-1) and Tie-2 [49]. On the contrary, Wnt5a downregulation or blocking inhibited EC proliferation and migration [49, 50]. In addition, tumor cell-derived Wnt5a was also involved in angiogenesis. For instance, melanoma cells released pro-angiogenic factor IL-6, vascular endothelial growth factor (VEGF) and MMP2, in a Wnt5a-dependent manner, and co-culture of Wnt5a-silenced melanoma cells with ECs showed decreased EC branching, compared with control melanoma cells [51].

**Figure 2:**
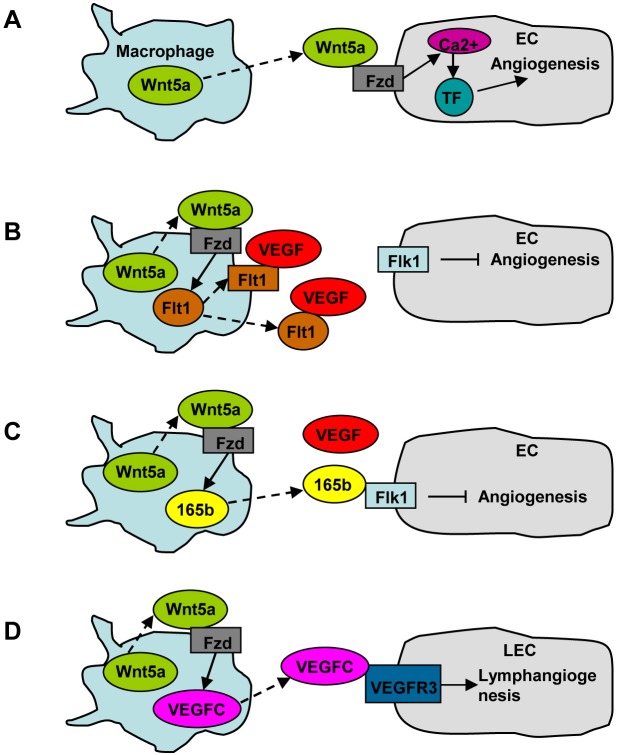
The role of macrophage-derived Wnt5a in angiogenesis and lymphangiogenesis **A.** Macrophage-derived Wnt5a induces angiogenesis by upregulating TF expression in ECs. **B.** Macrophage-derived Wnt5a inhibits angiogenesis by producing VEGF inhibitory receptor Flt1. **C.** Macrophage-derived Wnt5a inhibits angiogenesis by producing anti-angiogenic isoform of VEGF-A, VEGF-A165b. **D.** Macrophage-derived Wnt5a stimulates lymphangiogenesis by producing VEGF-C.

Angiogenesis is a character of both tumor development and tissue repair. Substantial evidence demonstrated that infiltrating macrophages attributed to tumor angiogenesis [52–54]. Macrophage recruitment in tumor microenvironment usually resulted from the expression of CCL2 by tumor cells [52–54]. Macrophages also promoted angiogenesis in some other pathological and physiological conditions. CD11b knockout mice exhibited impaired angiogenesis after partial hepatectomy [55]. Macrophages induced angiogenesis during liver fibrosis in an animal model treated with tetrachloride and bile duct ligation [56]. Moreover, PGC-1α-induced macrophage recruitment stimulated skeletal muscle angiogenesis [57]. As both Wnt5a and macrophages are implicated in angiogenesis, it is reasonable to speculate that macrophage-derived Wnt5a may stimulate angiogenesis. Indeed, it was observed that monocyte-secreted Wnt5a induced angiogenesis by upregulating tissue factor (TF) expression in ECs; this process depended on Fzd5, and involved Ca^2+^ signaling and NFκB pathway [58] (Figure 2A).

However, Wnt5a exhibits an inhibitory effect on angiogenesis in some other conditions. One mechanism is related to VEGF inhibitory receptor Flt1 (fms-like tyrosine kinase-1), also called VEGFR1, which has a higher affinity with VEGF than Flk1 (VEGFR2), but has limited signaling capacity (Figure 2B). Macrophage-derived Wnt5a induced Flt1 expression, which suppressed angiogenesis in postnatal retina and during wound repair [59, 60]. It was also shown that macrophage Raw264.7 expressed membrane and soluble Flt1 under the stimulation of exogenous Wnt5a [59]. Furthermore, macrophage-derived Wnt5a inhibited ischemia-induced angiogenesis by upregulating VEGF-A165b, an anti-angiogenic isoform of VEGF-A [61] (Figure 2C).

### Induction of lymphangiogenesis

Lymphangiogenesis is a character of both inflammation and cancer. It has been well accepted that macrophages are crucial for lymphangiogenesis in a various pathological or physiological conditions. VEGF-C/D from macrophages are the major contributors to lymphangiogenesis, especially under the action of inflammatory stimuli, such as LPS, IL-1β and TNF-α [62–65]. VEGF-C/D combined with VEGFR3 in lymphatic endothelial cell, initiating the signaling for lymphangiogenesis [66–68]. Overexpression of VEGF-C/D in tumor-associated macrophages (TAMs) attributed to lymphangiogenesis in tumors, which may promote tumor metastasis [65].

Evidence for the involvement of Wnt5a in lymphangiogenesis was provided by a study using Wnt5a knockout mice, which showed a reduction in the number of dermal lymphatic capillaries [69]. This study revealed the expression of Wnt5a and its receptor ROR1/2 and RYK in lymphatic endothelial cells (LECs), suggesting that LEC-derived Wnt5a may promote lymphangiogenesis in an autocrinal manner, just like EC-derived Wnt5a in promoting angiogenesis. It is unknown whether LEC-derived Wnt5a induces lymphangiogenesis via VEGF-C. The role of macrophage-derived Wnt5a in lymphangiogenesis was investigated recently using Wnt5a monocyte conditional knockout mice, which displayed reduced lymphangiogenesis in the corneas, accompanied by a reduction in VEGF-C expression [38] (Figure 2D).

## ROLES OF MACROPHAGE-DERIVED WNT5A IN HUMAN DISEASES

### Insulin resistance and diabetes

Animal studies have demonstrated that Wnt5a is an important player in insulin resistance and diabetes. Early evidence came from a study on SFRP5 knockout mice. SFRP5 is a Wnt5a antagonist, therefore, SFRP5 expression loss exaggerated the pro-inflammatory effects of Wnt5a, which was shown to be induced by obesity [44]. Administration of exogenous SFRP5 or ablation of JNK ameliorated insulin resistance, accompanied by a reduction in adipose tissue inflammation [44]. Similar results were observed in whole body Wnt5a knockout mice, which showed that Wnt5a ablation attenuated high-fat/high-sucrose (HFHS)-induced insulin resistance, and these mice displayed decreased expression of TNF-α, CCL2, and IL-6 in adipose tissue [43].

**Figure 3:**
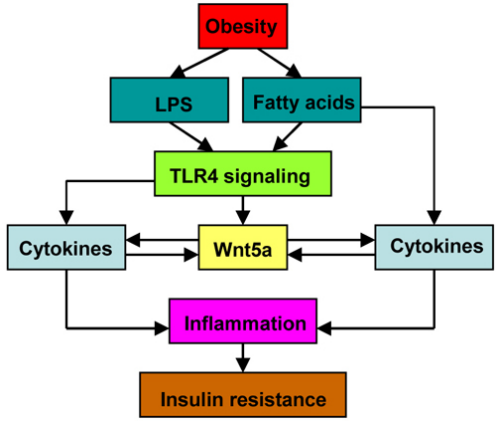
The involvement of macrophage-derived Wnt5a in adipose inflammation and insulin resistance Wnt5a acts as an “amplifier” in adipose tissue inflammation.

Study on myeloid Wnt5a knockout mice provided direct evidence that macrophage-derived Wnt5a mediated obesity-induced glucose metabolic dysfunction [43]. As expected, due to the partial ablation of Wnt5a expression, these mice displayed a milder phenotype than whole body Wnt5a knockout mice under HFHS [43]. Myeloid-restricted Wnt5a overexpression mice further confirmed the positive role of macrophage-derived Wnt5a in insulin resistance and diabetes. They exhibited enhanced pro-inflammatory cytokine production and glucose intolerance [43]. The mechanism behind the obesity-induced Wnt5a upregulation remains largely unknown. It may be attributed to metabolic entoxemia [70, 71] and increased level of fatty-acids [72, 73] induced by high-fat diet (Figure 3). As described previously, both LPS and pro-inflammatory cytokines are potent Wnt5a stimulators. Notably, LPS or TNF-α treatment also upregulated Wnt5a expression in adipocytes [31], indicating that adipocyte-derived Wnt5a is also implicated in adipose tissue inflammation and insulin resistance.

### Atherosclerosis

As an inflammatory disease, atherosclerosis is characterized by the accumulation of macrophages in the intima. Immunohistochemical analysis showed Wnt5a expression in human and murine atherosclerotic lesions, especially in the areas of macrophage infiltration, and was coincident with TLR-4 expression [74]. Wnt5a induction may be attributed to ox-LDL, which was shown to stimulate Wnt5a expression in human macrophages *in vitro* [33]. Wnt5a level was significantly higher in serum from atherosclerotic patients compared with healthy persons, and was correlated with the severity of atherosclerotic lesions [33, 74]. Furthermore, Wnt5a was involved in vascular calcification, a hallmark of advanced atherosclerosis [75]. Importantly, anti-Wnt5a treatment inhibited atherosclerotic development in apolipoprotein E-deficient (ApoE−/−) mice fed a high-fat diet, accompanied by reduced expression of inflammatory cytokines [76].

### Cancer

Wnt5a displays multifunctional in human malignancies. Wnt5a loss was found in leukemias [77, 78], ovarian cancers [79], and thyroid carcinomas [80]. In these malignancies, Wnt5a acted as a tumor suppressor mainly via antagonizing carcinogenetic Wnt/β-catenin pathway. However, in other human tumors, such as melanoma and pancreatic cancer, Wnt5a increased tumor cell invasion and migration through actin reorganization, cell adhesion and epithelial to mesenchymal transition (EMT) [81–83]. These studies indicate that Wnt5a may inhibit β-catenin-related carcinogenesis, but promote tumor progression via β-catenin-independent mechanisms. The exact role of Wnt5a in human cancers depends on the tumor context.

As macrophages are a source of Wnt5a, it is interesting to explore the role of TAM-derived Wnt5a in human malignancies. Wnt5a expression was observed in TAMs in human breast cancers [34] and colon cancers [84]. Coculture experiments showed that tumors cells induced Wnt5a expression in macrophages [34–36]. Some particles from breast cancer cells were responsible for the upregulation of Wnt5a in macrophages [36]. Conversely, macrophage-derived Wnt5a promoted breast cancer cell invasiveness [34] and gastric cancer cell migration [27]. The reciprocal interaction between tumor cells and macrophages do not always promote tumor progression. It was observed that macrophage-derived Wnt5a induced BCC cell differentiation and regression [35].

## CONCLUSIONS

**Figure 4:**
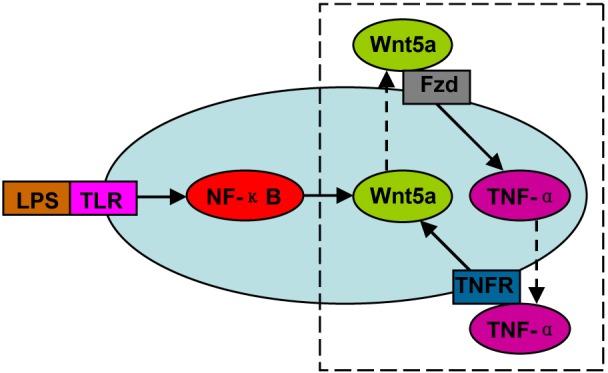
The positive feedback loop between Wnt5a and inflammatory cytokines (e.g. TNF-α) This loop can be triggered by LPS-TLR-NF-κB signaling, and amplify the inflammatory responses.

Consistent with the characterized infiltration of macrophages in inflammation and cancer, macrophage-derived Wnt5a has been proved to be involved in human inflammatory diseases and cancers, mainly by inducing pro-inflammatory cytokine release, angiogenesis and lymphangiogenesis. A positive feedback loop exists between Wnt5a and inflammatory cytokines, supported by the finding that inflammatory cytokines are also potent inducers of Wnt5a (Figure 4). This loop may be triggered by LPS-TLR-NF-κB signaling, and aggravate the inflammatory responses. These findings clearly demonstrate that macrophage-derived Wnt5a should be considered as a target in the treatment of these diseases.

LPS induced Wnt5a production by macrophages via TLR-NF-κB signaling, and Wnt5a signaling inhibition reduced IFN-β and IFN-γ production and CD14 expression by macrophages, suggesting that Wnt5a may be a component in macrophage innate immunity [24]. Inconsistently, although Wnt5a-Fzd5 signaling enhanced macrophage phagocytosis, it was not accompanied by an increase in bacteria killing, and on the contrary, Wnt5a inhibitor IWP-2 reduced macrophage phagocytosis, but increased bacterial killing [40]. These finding indicate that more investigations are needed to explore the exact role of macrophage-derived Wnt5a in infection and sepsis.

Tumor cell-derived Wnt5a has been demonstrated to play a dual role in human cancer initiation and progression. However, few studies have focused on the exact role of TAM-derived Wnt5a. Does TAM-derived Wnt5a promote cancer cell migration by inducing angiogenesis and lymphangiogenesis? Does TAM-derive Wnt5a trigger local inflammation in the tumor microenvironment? Actually, inflammation has been considered as a hallmark of cancer. And how does TAM-derive Wnt5a regulate TAM immune function? A reasonable speculation is that Wnt5a may in part mediate the action of TAMs on cancers, and more studies are needed to answer these questions.
